# Graph Node Classification to Predict Autism Risk in Genes

**DOI:** 10.3390/genes15040447

**Published:** 2024-04-01

**Authors:** Danushka Bandara, Kyle Riccardi

**Affiliations:** Department of Computer Science and Engineering, Fairfield University, Fairfield, CT 06824, USA; kyle.riccardi@student.fairfield.edu

**Keywords:** autism risk classification, graph neural networks, gene networks, chromosome band features

## Abstract

This study explores the genetic risk associations with autism spectrum disorder (ASD) using graph neural networks (GNNs), leveraging the Sfari dataset and protein interaction network (PIN) data. We built a gene network with genes as nodes, chromosome band location as node features, and gene interactions as edges. Graph models were employed to classify the autism risk associated with newly introduced genes (test set). Three classification tasks were undertaken to test the ability of our models: binary risk association, multi-class risk association, and syndromic gene association. We tested graph convolutional networks, Graph Sage, graph transformer, and Multi-Layer Perceptron (Baseline) architectures on this problem. The Graph Sage model consistently outperformed the other models, showcasing its utility in classifying ASD-related genes. Our ablation studies show that the chromosome band location and protein interactions contain useful information for this problem. The models achieved 85.80% accuracy on the binary risk classification, 81.68% accuracy on the multi-class risk classification, and 90.22% on the syndromic classification.

## 1. Introduction

Autism spectrum disorder (ASD) is a deficit of social communication or sensory-motor function based on genetic association and other causations [1]. The genetic association is supported by the inheritance rate observed by Tick et al.’s [2] meta-data analysis on twins, which determined the inheritance of ASD to range between 64 and 91 percent. Tick et al. also associated a 0.98 correlation between genetics and neurodevelopmental disorders. De novo mutations further express the relationship inheritance has on ASD because these genetic mutations happen specifically with stem cell divisions and maturation of the female gametes within the trio (father, mother, and child) [3]. These genetic mutations are based on mutations found between the trio, determining the de novo mutation that carries the high inheritance seen within the Tick et al. analysis [4,5,6].

In the *Handbook of Clinical Neurology* [7], ASD connection is associated with an estimated 1000 genes determined based on genetic linkage between chromosome location (loci) and possible genetic risk. Alarcón et al. [8] performed a comprehensive study on chromosome regions 7q and 17q, finding that region 7q2-35 has a strong possibility of associating with ASD while also noting that other areas, like chromosome 3q, might also have an association with ASD. Copy number variants (CNV) is the process of adding, removing, or copying portions of deoxyribonucleic acid (DNA) [9]. CNV shows the exact genetic correlation on specific regions of the chromosome band, further exemplifying the link between loci and ASD.

Another genetic association is a common variant which significantly affects ASD risk. Grove et al. [10] used a genome-wide association study that determined common variants’ strong, robust connection with ASD. Common variants are significant factors in most diseases, as they relate to 90 percent of the differences between individuals [11]. From an individual perspective, common variants create minimal significance, but by putting all the variants together, we determine a noticeable impact for risk [12]. Common variants make up 40–60 percent of the risk factor when evaluating ASD [13].

Between all of the variants and genetic mutations, we see that these mutations and variants are connected within a network. Kolchanov et al. describe gene networks as a group of genes functioning in a coordinated manner [14]. As seen, common variants, de novo variants, and CNV are all the byproducts of genes functioning in coordination with one another, and that function creates variants with high ASD risk. These variants all combined create a gene network that links all of these variants together, showing us the association/non-association of a gene [15]. Graph neural networks have recently been proposed to address classic yet challenging graph problems, such as node or graph classification, and have been used for graph structure learning and graph classification. The diverse applications of GNNs in these studies underscore their broad utility in addressing various problems across various domains, including social networks, healthcare, and other network structures [16,17,18,19]. Our proposed experiment uses these gene networks and graph neural networks to determine if a gene has an association and the level of risk associated with the gene. The specific graph neural network models we use are Nodeformer [20], Graph Sage [21], and graph convolutional network (GCN) [22]. Within this experiment, we will use a binary and multi-class classifier to predict the likelihood of a gene being associated with autism risk. We will also use another binary classification approach to predict whether a gene is associated with a particular syndrome related to ASD. In addition, we conduct extensive experiments to determine the effect of gene location and gene network information on the classification.

## 2. Related Works

Genome-wide association study (GWAS) is a method used to find genetic variants that are associated with a particular disease or trait. It does this by comparing the DNA of people with the disease or trait to the DNA of people who do not have the disease or trait. Bralten et al. [23] used GWAS to find a connection between genetic sharing between ASDs and the autistic traits ‘childhood behavior’, ‘rigidity’, and ‘attention to detail’. Grove et al. [24] used this technique to find five genome-wide significant loci associated with autism risk. Krishnan et al. [25] developed a machine learning approach based on a human brain-specific gene network to present a genome-wide prediction of autism risk genes, including hundreds of candidates for which there is minimal or no prior genetic evidence. Rahman et al. (2020) [26] established a network known as a brain tissue-specific Functional Relational Network (FRN), which applies machine learning techniques to predict the genomic-activated autism-related genes. Furthermore, Ismail et al. (2022) [27] proposed a hybrid ensemble-based classification model for predicting ASD genes using machine learning. Lin et al. [28] employed a machine learning-based approach to predict ASD genes using features from spatiotemporal gene expression patterns in the human brain, gene-level constraint metrics, and other gene variation features. Furthermore, Brueggeman et al. [29] utilized machine learning and genome-scale data to forecast autism gene discovery.

Genes that confer risk for ASD are likely to be functionally related, thus converging on molecular networks and biological pathways implicated in disease [30]. Taking this idea further, Krumm et al. [31] showed that ASD genes with de novo mutations converged on pathways related to chromatin remodeling and synaptic function. Some later studies showed that integrating known risk genes using a protein–protein interaction (PPI) network can identify novel genes involved in ASD [32,33]. In this paper, we are analyzing the risk assessment using binary risk association labels, multi-class risk association labels (based on a score of confidence), and binary syndromic association for whether the gene is associated with an overarching medical condition.

### Use of Graph Neural Networks to Predict Disease

In machine learning, many techniques have been used to predict diseases using gene networks, risk assessment ASD, and overall disease risk discovery using machine learning techniques. The first is the interaction discovery made in protein–protein interaction networks by Fenq et. al, who discovered using omic data that they can determine new links with these interaction networks [34]. Wang et al. used attention-based graph neural networks to identify ASD based on the activity within brain scans [35]. Beyreli et al. created DeepND, a multitask graph convolutional network that used an autism network and an intellectual disability network to determine the risk for both [36]. They achieved a median AUC of 87%. Lu et al. used a graph neural network and a patient network to classify whether a patient suffers from a chronic illness or not [37]. Wang and Avillach used DeepAutism, a convolutional network designed to diagnose ASD based on the presence of high-risk gene variants [38]. They achieved 88.6% accuracy. Motsinger et al. used gene–gene interaction networks and neural networks to classify the risk people carry for Parkinson’s disease [39]. Laksshman et al. created deep bipolar, which specializes in identifying gene mutations to determine whether somebody is bipolar [40].

## 3. Methodology

### 3.1. Dataset

The datasets used for these experiments were the Sfari dataset [41] and protein interaction network (PIN) data [15]. The Sfari dataset contains gene associations and rankings (labels). It also contains the chromosome band location of each specific gene. For our binary risk association and multi-class risk association classification, we used the confidence score on Sfari, which ranks from most confident to least confident association in ASD (ranking from 1 to 3).

- Case (1) Binary risk association classification. If a gene is contained in the SFARI dataset, it is automatically considered to contain risk for ASD. The two classes for this are as follows:
  1 Gene with associated risk;
  2 Gene without associated risk.
- Case (2) The multi-class risk association classification uses three risk levels for its labels. The classes for this classification are as follows:
  1 No gene association;
  2 Low gene association;
  3 Moderate gene association;
  4 High gene association.
- Case (3) Syndromic gene classification. (Syndromes are collections of multiple, related medical signs and symptoms that occur together. Mutations in a syndromic gene can lead to a variety of problems affecting different parts of the body and causing a recognizable pattern of symptoms.) The syndromic risk association shows us the identification of syndromic and possible non-syndromic genes. (Sfari dataset lists all specifically syndromic gene associations). The classes are as follows:
  1 Syndromic gene;
  2 Non-syndromic gene.

The PIN dataset comprises data on protein–protein interactions, elucidating which proteins interact. These interactions can be represented as a network or graph, where proteins are nodes, and interactions between them are edges or connections. The PIN dataset contains all protein–protein interactions both associated and not associated with ASD.

### 3.2. Preprocessing

The first preprocessing step is to filter out anything in the PIN dataset that is not specified as being a human gene interaction. Next, we add the chromosome band location and labels (binary, multi-class, and syndromic) from the Sfari dataset to the genes in the PIN dataset to have our edges and associated labels. The chromosome band locations obtained from the Sfari dataset are used as node features. Equation (1) shows the definition of the graph:

(1) G=(V,E,C)

- *G*: The graph representing genes and their interactions.
- *V*: The set of nodes, where each node represents a gene and is associated with its location (feature).
- *E*: The set of edges representing interactions or connections between genes in the graph.
- *C*: The set of labels representing autism risk for each gene, where each gene node is associated with a label indicating its classification (high confidence, strong candidate, suggestive evidence).

**Figure 1:**
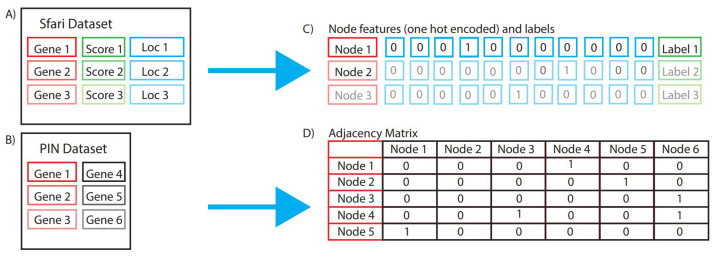
(**A**) Sfari dataset format, (**B**) PIN dataset format, (**C**) nodes, node features, and node labels generated using Sfari dataset, (**D**) adjacency matrix created using PIN dataset.

The chromosome band is placed in a one-hot encoder that splits our feature into a list of 472 binary features representing every possible location where the gene has been observed. This is then also followed up with using our edge list to create an adjacency matrix and then passing our labels. In the case of multi-class classification, the high confidence class contains 214 genes, the strong candidate class includes 530 genes, and the suggestive evidence class has 69 genes. This is paired with 11,403 no associated genes, which shows a severe imbalance. To fix this imbalance, we upsample [42] all of the genes to ensure we have a balanced classification among all four classes. For the binary class, we take the high confidence class, strong candidate class, and the suggestive evidence class together, which is 813 genes with the same paired 114,03 non associated genes. Lastly, the syndromic labels are split into 169 syndromic genes and 12,047 that are not syndromic. The preprocessing steps are visualized in Figure 1.

### 3.3. Models

The models used for this experiment are graph convolutional network [22], Graph Sage [21], and Multi-Layer Perceptron (MLP). The models each contain two layers, each having a rectified linear unit (ReLu) between the layers and ending with a multilayer perceptron (MLP). These models are set up to use the adjacency matrix, feature matrix, and selected labels to create either a binary or multi-class classification.

### 3.4. Graph Convolutional Network

GCN uses the convolutional properties of a graph network to facilitate a connection of both the features and the network. This process is performed in a layer of the GCN called the propagation Layer (Equation (2)), where l is the layer of the GCN operation; $h_{v}^{\left(l\right)}$ is the feature vector of node v in the l-th layer; N(v) is the set of neighboring nodes of node v; $W^{\left(l\right)}$ is the weight matrix for the l-th layer; $c_{v}$ is a normalization factor for node v; and σ is an activation function (e.g., ReLU):

(2) $$\begin{array}{c} h_{v}^{\left(l+1\right)}=\sigma \left(\sum_{u\in N(v)}\frac{1}{c_{v}}W^{\left(l\right)}h_{u}^{\left(l\right)}\right) \end{array}$$

GCN is more of a top-down approach, which looks at the entire picture of the network and its feature matrix for performing calculations. This model ignores the effect a singular node has on the network but instead looks at all of the interactions together through matrix multiplication. Once this is done, we obtain our embedding matrix, allowing us to classify what class a node belongs to.

### 3.5. Graph Sage

Graph Sage takes an aggregation of all neighboring nodes in the network. The Graph Sage operation is defined in Equation (3), where l is the layer of the GraphSAGE operation; $h_{v}^{\left(l\right)}$ is the feature vector of node v in the l-th layer; N(v) is the set of neighboring nodes of node v; $W^{\left(l\right)}$ is the weight matrix for the l-th layer; AGGREGATE is the aggregation function (e.g., mean, sum, or LSTM-based aggregation); and σ is the activation function (e.g., ReLU):

(3) $$\begin{array}{c} h_{v}^{\left(l+1\right)}=\sigma \left(W^{\left(l\right)}\cdot \mathrm{AGGREGATE}\left(h_{u}^{\left(l\right)},\forall u\in N\left(v\right)\right)\right) \end{array}$$

Mean aggregated features are then concatenated to create new features for every node in the feature matrix. This model infers the connection of neighboring nodes. This connection creates another approach but an essential method for interpreting graph networks. Instead of using a broad look like GCN, this takes a neighboring approach, which instead infers the connection a feature relationship that not only the current node but its neighboring nodes have with each other.

### 3.6. Graph Transformer (Nodeformer)

NodeFormer [20] is a graph transformer model known for its all-pair attention mechanism, enabling efficient graph data processing. Traditional graph neural networks propagate signals over a sparse adjacency matrix, while graph transformers [43] can be seen as propagating signals over a densely connected graph with layer-wise edge weights. The latter requires estimation for the N*N attention matrix and feature propagation over such a dense matrix. At each layer of the graph transformer, the embeddings of the current layer are mapped to query, key, and value vectors. Then, all pair attention is calculated for aggregating the features. Equation (4) shows how the attention operation is applied to node u:

(4) $$\begin{array}{c} q_{u}^{\left(k\right)}=W_{Q}z_{u}^{\left(k\right)},k_{u}^{\left(k\right)}=W_{K}z_{u}^{\left(k\right)},v^{\left(k\right)}=W_{V}z_{u}^{\left(k\right)}\\ z_{u}^{\left(k+1\right)}=\sum_{v=1}^{N}\frac{\exp\left({\left(q_{u}^{\left(k\right)}\right)}^{⊤}k_{v}^{\left(k\right)}\right)}{\sum_{w=1}^{N}\exp\left({\left(q_{u}^{\left(k\right)}\right)}^{⊤}k^{\left(k\right)}\right)}v_{v}^{\left(w\right)} \end{array}$$

z denotes the node embeddings, and q, k, and v denote the query, key, and value vectors. The $W_{Q}$, $W_{K}$, and $W_{V}$ are learnable weights at the k-th layer. The all-pair attention operation in Equation (4) has O(N) complexity. However, when it gets applied to all nodes in the graph, its complexity explodes to $O(N^{2})$. To avoid this, Nodeformer decouples the summation operation from the dot product as shown in Equation (5). In this approach, the summations are independent of the node u. Therefore, the summations can be precomputed in O(N) complexity and then shared among all nodes. The layer embedding calculation will thus be of O(N) complexity:

(5) $$z_{u}^{\left(l+1\right)}=\sum_{v=1}^{N}\frac{\phi {\left(q_{u}\right)}^{⊤}\phi \left(k_{v}\right)}{\sum_{w=1}^{N}\phi {\left(q_{u}\right)}^{⊤}\phi \left(k_{w}\right)}\cdot v_{v}=\frac{\phi {\left(q_{u}\right)}^{⊤}\left(\sum_{v=1}^{N}\phi \left(k_{v}\right)\cdot v_{v}^{⊤}\right)}{\phi {\left(q_{u}\right)}^{⊤}\sum_{w=1}^{N}\phi \left(k_{w}\right)}$$

## 4. Experiments

### 4.1. Baseline Model

To obtain a baseline performance for the dataset in each of the three cases mentioned above, we use a vanilla MLP in each case without the use of network information. We use the same training and test data and parameters for the baseline as the rest of the models.

### 4.2. Node Features Ablation Study

As an ablation study, the three classifiers are run featureless (without node features). This allows the evaluation of the importance of node features for the performance of our models. If the classification performance decreases when the features are removed, that would support the claim that the node features provide useful information to the models.

### 4.3. Model Architectures

**Figure 2:**
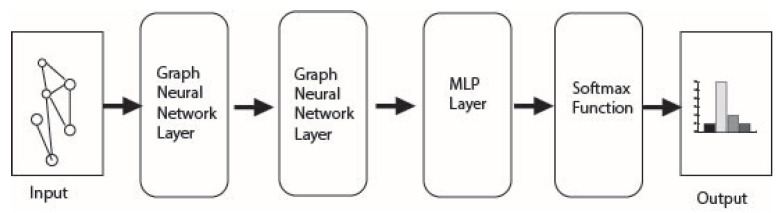
Model structure of the MLP, GCN, and Graph Sage models.

**Figure 3:**
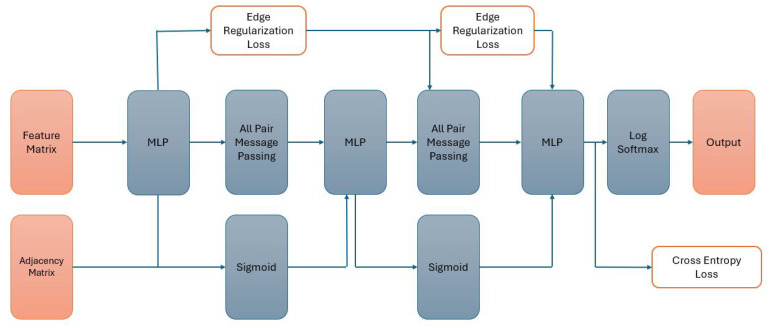
Model structure of the graph transformer model.

The experiment uses three configurations for each of the four classifiers: (1) two GCN layers attached to an MLP layer, (2) two Graph Sage layers attached to an MLP layer, (3) two graph transformer layers attached to an MLP layer, and (4) three MLP layers. The models are fed the adjacency matrix, feature matrix, and labels (binary or multi-class). The adjacency matrix considers all gene interactions within the PIN dataset and gathers its location and labels through the SFari dataset. The model structure used in the (1), (2) and (3) configurations is shown in Figure 2. Model structure for configuration (4) is shown in Figure 3.

The results of the multi-class model are evaluated using specificity and sensitivity for all four classes, accuracy, and F1 Score for the test data. The test splits are 25 percent of the original dataset and split after Graph SMOTE [42] upsampling to balance the labels. The process of upsampling not only requires us to add a row to our feature matrix to account for the new nodes but also requires us to add a row and column to our adjacency matrix to account for the new node as well. The process of adding a row to the feature matrix and a column and row to the adjacency matrix is performed for each gene selected for duplication in the upsampling. The experiment is conducted using the hyperparameters listed in Table 1.

## 5. Results

### 5.1. Binary Risk Association Classification Test Results

Table 2 displays the performance metrics of the four models employed in the binary risk association classification task. GCN demonstrates exemplary performance in specificity, achieving a perfect score of 1.00, indicating its robustness in correctly identifying true negatives. However, its sensitivity is 0.69, suggesting a relatively lower capacity to detect true positives correctly. On the other hand, the Graph Sage model displays a commendable balance between specificity (0.94) and sensitivity (0.82), showcasing its ability to discern both true negatives and true positives effectively. The MLP baseline model also exhibits competitive performance, with specificity at 0.92 and sensitivity at 0.70, positioning it between the other two models regarding overall performance metrics.

Table 3 displays the performance metrics of four distinct models employed in the binary risk association classification task. Graph Sage emerges as the top performer, exhibiting the highest F1 Score of 0.87 and an accuracy of 85.80%. The graph transformer model also demonstrates competitive performance, achieving an F1 Score of 0.75 and an accuracy of 75.01%. The Featureless GCN, Graph Sage, and graph transformer model versions present lower performance metrics.

**Figure 4:**
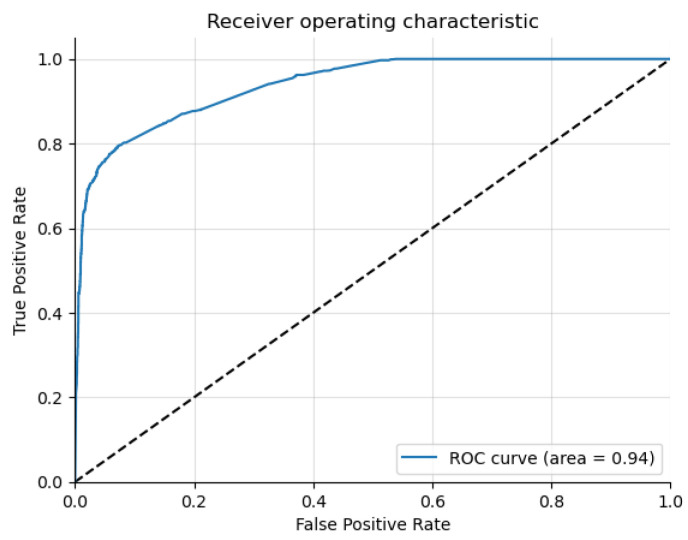
The receiver operating characteristic curve for the binary risk association classification using the Graph Sage model.

Figure 4 is the receiver operating characteristic curve that shows the optimal threshold for classification on the test dataset. The Graph Sage model is used here and in subsequent ROC curves and t-SNE plots due to its higher performance compared to the other models.

**Figure 5:**
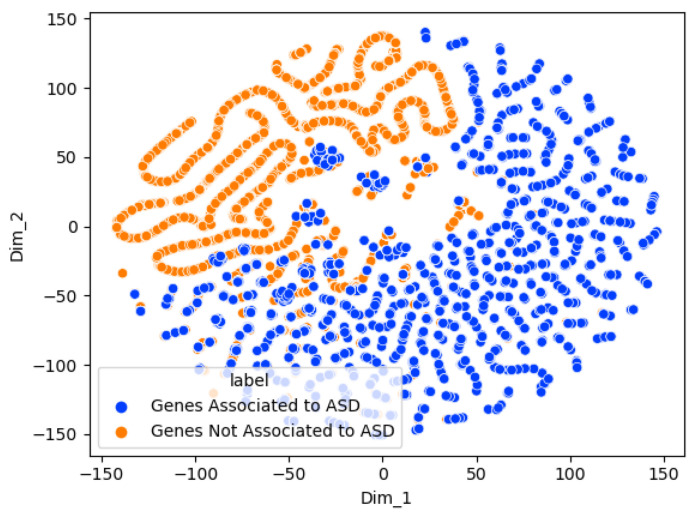
Visualization of the Graph Sage model genes using t-distributed stochastic neighbor embedding (t-SNE). Different colors represent different classes.

Figure 5 shows a t-SNE chart of the Graph Sage binary risk classifier. This method allows the high-dimensional data of the graph nodes (genes) to be reduced to two for visualization while preserving the data’s local structures. The t-SNE chart shows two clear clusters for the genes associated and not associated with ASD. This indicates that the two groups are distinguishable using the Graph Sage model.

### 5.2. Multi-Class Risk Association Test Results

Table 4 presents specificity and sensitivity metrics for four distinct association classes in the multi-class classifier, comparing the performance of GCN, Graph Sage, graph transformer, and MLP (baseline). Across association levels—‘No Association’, ‘Low Association’, ‘Moderate Association’, and ‘High Association’—GCN consistently demonstrates high specificity, particularly excelling in identifying ‘No Association’ and ‘High Association’ classes. Graph Sage performs well in various classes, showcasing balanced sensitivity, especially notable in ‘Low Association’ and ‘High Association’. Graph transformer and MLP display lower performance.

As seen in Table 5, Graph Sage is the best performer in our test. Graph Sage obtains the highest F1 Score of 0.83 and an accuracy of 81.68%. Graph transformer achieves the second highest performance. GCN fails to achieve the performance of the baseline MLP model. The Featureless versions of the models exhibit lower performance metrics, with the Graph Sage Featureless model obtaining an F1 Score of 0.32 and an accuracy of 43.44%, and the GCN Featureless model achieving an F1 Score of 0.27 and an accuracy of 43.24%, which is far below the baseline. Thus, the graph structure and node features seem to positively impact classifier performance.

Table 6 contains the top 10 list for each class of genes based on the highest confidence.

**Figure 6:**
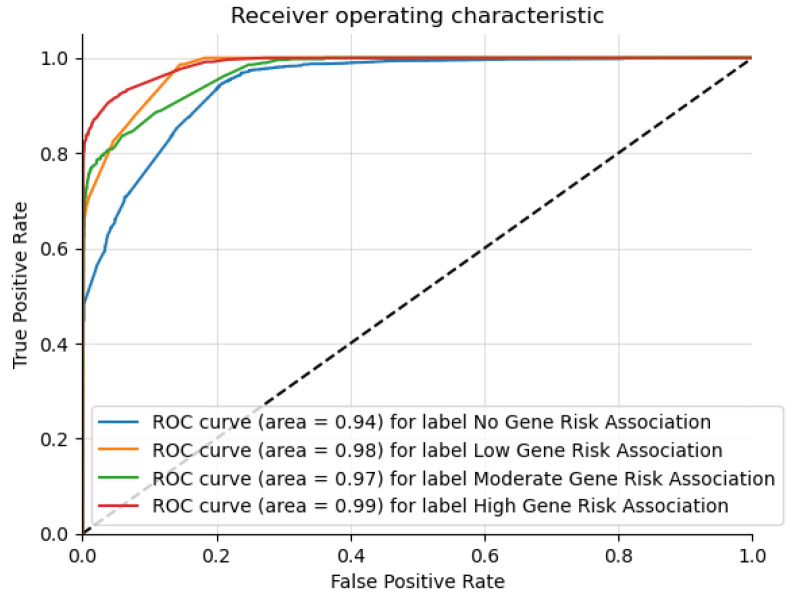
The ROC curve for multi-class risk association classification.

Figure 6 shows the receiver operating characteristic curves for each risk class. From the chart, it is evident that the area under the curve values are similarly high for each of the classes, showing that the Graph Sage multi-class model has a good classification ability across all the risk levels.

**Figure 7:**
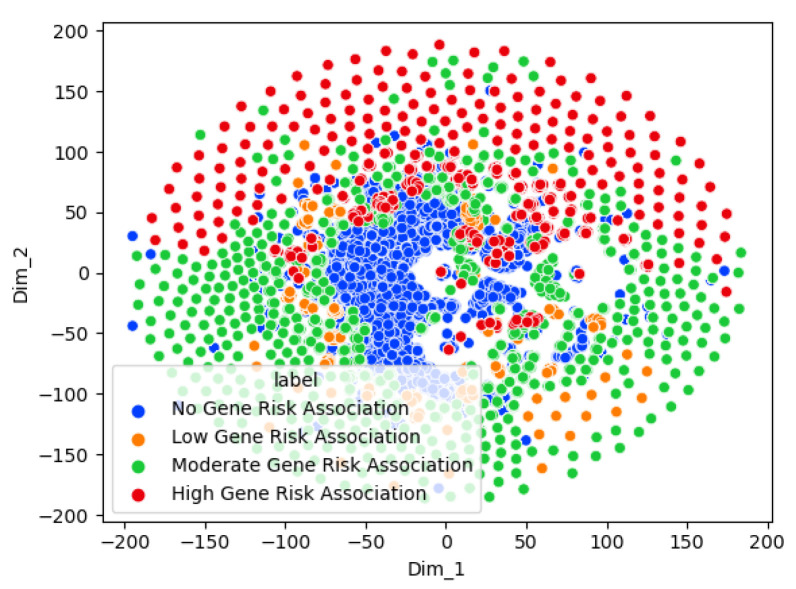
Visualization of the Graph Sage multi-class classifier genes using t-distributed stochastic neighbor embedding (t-SNE). Different colors represent different classes.

Figure 7 shows a t-SNE chart of the Graph Sage multi-class risk classifier. The t-SNE chart shows a clear separation between the high- and low-risk genes. At the same time, the moderate-risk genes are spread between the high- and low-risk genes. This agrees with the intuition that the ASD risk is a spectrum ranging from low to high. The non-associated genes are tightly clustered and separate from the other genes. This shows that the model can discern between no risk association and the other risk levels.

### 5.3. Syndromic Test Results

Table 7 shows that GCN exhibits perfect specificity at 1.00, denoting its capability to accurately identify true negatives while achieving a sensitivity of 0.68, indicating a lowered capacity in correctly detecting true positives. Graph Sage demonstrates high performance with specificity at 0.96 and sensitivity at 0.90, effectively showing its ability to discern both true negatives and true positives. The baseline MLP model shows comparable performance to GCN. Graph transformer displays balanced performance.

Table 8 shows that Graph Sage performs the best, exhibiting the highest F1 Score of 0.90 and an accuracy of 90.22%. The MLP model shows lower performance with an F1 Score of 0.86 and an accuracy of 86.39%. In contrast, the Featureless versions of GCN, Graph Sage, and graph transformer demonstrate much lower performance metrics, with the Graph Sage Featureless model attaining an F1 Score of 0.51 and an accuracy of 46.71%. The results are in line with what we observed from the binary risk classification.

Table 9 shows the top 10 genes for each class. These genes are ranked based on their confidence levels within their respective classes, offering insights into potential associations or relevance to syndromic and non-syndromic conditions. From the list of syndromic genes, many of them are associated with brain development or neuronal development syndromes (TRIP12 [44], NSD1 [45], CTNND2 [46], CADPS2 [47], MEF2C [48], SOX5 [49], and GRIP1 [50]). In contrast, most non-syndromic genes do not have a clear connection with the brain or neural development. This indicates that the model can differentiate between ASD-related and non-related genes.

**Figure 8:**
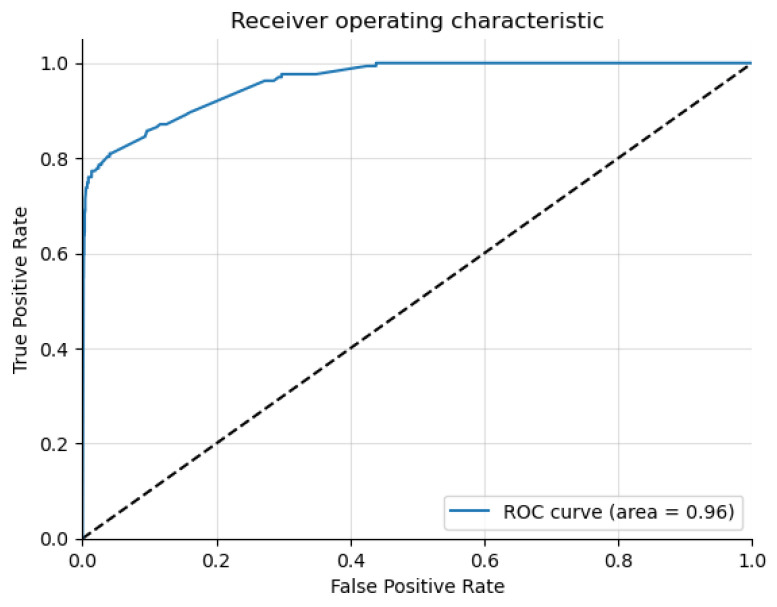
The receiver operating characteristic curve for syndromic classification.

Figure 8 shows the receiver operating characteristic curve for the syndromic classification.

**Figure 9:**
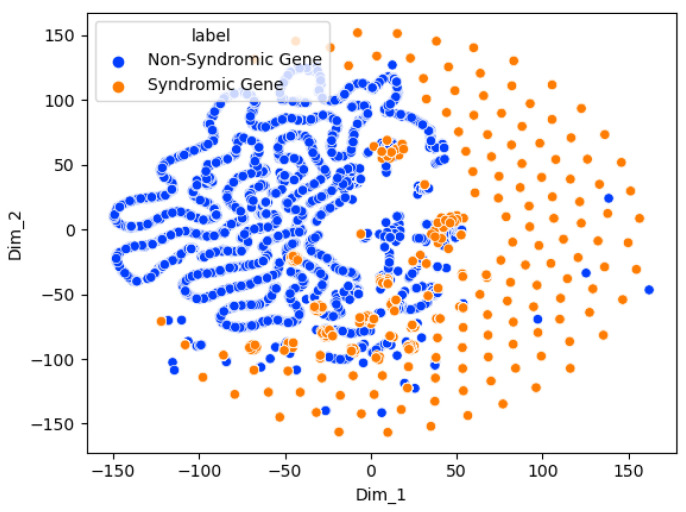
Visualization of the Graph Sage syndromic classifier genes using t-distributed stochastic neighbor embedding (t-SNE). Different colors represent different classes.

Figure 9 shows that the syndromic and nonsyndromic genes form two separate clusters, thus showing the ability of the model to distinguish the two classes.

## 6. Discussion

The results highlight that Graph Sage surpasses the baseline model in every case. The GCN surpasses the baseline in most cases but not all. This implies that while graph structures offer valuable information for the three classification problems, their utility may differ in specific contexts. The graph transformer model does not surpass the Graph Sage model in our experiments. It remains to be seen whether a larger dataset will improve the graph transformer model performance beyond the other models. The featureless models perform significantly worse than their counterparts in every case. This shows that the node features (Chromosome band locations) also contribute heavily to the classification. From the t-SNE charts, we can see clear clusters forming for each of the classes. In the case of multi-class risk association, the moderate-risk genes seem to fall in between the high-risk and low-risk genes. This shows that the risk levels are a spectrum ranging from low to high. Analyzing the top genes shows that the ASD risk-associated genes discovered by the models tend to be genes connected to brain or neuronal development. This further shows the ability of the models to distinguish the risk levels. However, further study of the top genes is needed before we can confirm this.

Limitations of our study can stem from the source datasets. For example, autism datasets such as Sfari tend to contain data from clinical populations, and thus they could exclude individuals with mild levels of autism. Therefore, our models could also be biased towards severe autism levels. Another potential source for bias is the under-representation of certain groups such as minority groups from the datasets. This could affect the classification accuracy of models when it comes to those populations. The benefits of our approach include the ability for early identification and intervention. In the case of autism, such early interventions can improve therapy outcomes. Research has shown that involving pediatricians as initial diagnosticians in multidisciplinary evaluations for young children with ASD [51]. The biomarkers identified by our method could be used alongside other behavioral and brain imaging techniques as a comprehensive early diagnosis workflow. Since our method has good explainability through predictive features and t-SNE visualizations, it gives confidence to doctors and patients when used in a diagnostic setting.

Our method also holds promise for targeted therapeutic interventions for individuals with autism. Precision medicine approaches can be developed to target underlying genetic mechanisms contributing to ASD. This may include pharmacological interventions aimed at modulating neurotransmitter systems or gene therapy strategies to correct genetic abnormalities.

## 7. Conclusions

The experiment demonstrates the efficacy of graph neural networks in assessing the risk of gene association with ASD and identifying whether a gene is syndromic. Specifically, our findings highlight Graph Sage as particularly promising in uncovering correlations related to ASD. This success suggests broader applications of this model in diverse ASD-related domains, potentially including specific disorders within the autism spectrum. Moreover, the results indicate that leveraging networks can notably enhance model performance, motivating further exploration into more advanced graph neural network models. This groundwork lays a strong foundation for developing a robust tool to identify genes associated with neurological disorders.

## Figures and Tables

**Table 1:** Parameters used for training the models.

Parameter Name	Value
Learning rate	0.001
Weight decay	$5\times 10^{-4}$
epsilon	$1\times 10^{-4}$
Batch size	64
Epoch	5000
Output Function	Log Softmax
Activation Function	reLu
Optimizer	AdamW

**Table 2:** The performance of the four models in the binary risk association classification.

Model Name	Specificity	Sensitivity
MLP	0.92	0.70
GCN	**1.00**	0.69
Graph Sage	0.94	**0.82**
Graph T/F	0.76	0.74

**Table 3:** The F1 Score and accuracy for all five binary graph neural network models.

Model Name	F1 Score	Accuracy
GCN	0.73	77.08
Graph Sage	**0.87**	**85.80**
Graph T/F	0.75	75.01
MLP	0.78	78.88
GCN Featureless	0.72	63.57
Graph Sage Featureless	0.41	66.61
Graph T/F Featureless	0.29	46.36

**Table 4:** The specificity and sensitivity for all four classes in the multi-class graph neural network model.

Class Number	GCN	Graph Sage	Graph Transformer	MLP
No Association	1.00/0.94	0.95/0.80	0.83/0.78	0.96/0.76
Low Association	1.00/0.76	0.94/1.00	0.95/0.88	0.90/0.89
Moderate Association	1.00/0.61	0.98/0.80	0.96/0.73	0.97/0.65
High Association	1.00/0.99	0.98/0.89	0.97/0.90	0.97/0.75

**Table 5:** The F1 Score and accuracy for all five multi-class graph neural network models.

Model Name	F1 Score	Accuracy
MLP	0.72	69.80
GCN	0.56	55.19
Graph Sage	**0.83**	**81.68**
Graph T/F	0.79	78.13
GCN Featureless	0.27	43.24
Graph Sage Featureless	0.32	43.44
Graph T/F Featureless	0.20	26.01

**Table 6:** The highest confidence level genes as predicted by the Graph Sage multi-class model for the various risk levels.

High Association	Moderate Association	Low Association
FMR	TOP3B	PPP3CA
TCF	ELAVL2	TCEAL1
HRA	HTR3C	HCN1
CUL	KHDRBS2	IKZF1
PTE	RBFOX1	BAIAP2L1
CHD	CTNND2	TRPC5
NLGN	PON1	YWHAZ
CTNNB	PCDH10	CACNB1
KMT2	PLN	CACNB3
NSD	CNTNAP4	PC

**Table 7:** The specificity and sensitivity for all four of our models in the syndromic classification.

Model Name	Specificity	Sensitivity
MLP	0.97	0.75
GCN	**1.00**	0.68
Graph Sage	0.96	**0.90**
Graph T/F	0.93	0.82

**Table 8:** The F1 Score and accuracy for all five syndromic classifiers.

Model Name	F1 Score	Accuracy
MLP	0.86	86.39
GCN	0.78	78.66
Graph Sage	**0.90**	**90.22**
Graph T/F	0.78	76.46
GCN Featureless	0.72	48.16
Graph Sage Featureless	0.51	46.71
Graph T/F Featureless	0.29	43.04

**Table 9:** The top 10 genes based on confidence for both syndromic and non-syndromic classes.

Non-Syndromic	Syndromic
CDK11B	TRIP12
CCL21	TNRC6B
MATR3	NSD1
DCX	CTNND2
ATXN1	CADPS2
HOXB13	MST1R
MIR101-1	TSPAN7
WNT1	MEF2C
C4ORF6	SOX5
CCDC27	GRIP1

## Data Availability

The dataset used in this study is derived from publicly available datasets. As such, the authors will provide the code required to generate the dataset in a public repository.
